# 5-HT is a potent relaxant in rat superior mesenteric veins

**DOI:** 10.1002/prp2.103

**Published:** 2015-01-05

**Authors:** Stephanie W Watts, Emma S Darios, Bridget M Seitz, Janice M Thompson

**Affiliations:** Department of Pharmacology and Toxicology, Michigan State UniversityEast Lansing, Michigan, 48824

**Keywords:** 5-HT, hypotension, venous circulation

## Abstract

Serotonin (5-HT, 5-hydroxytryptamine) reduces blood pressure of the conscious rat when administered chronically (1 week). 5-HT does not directly relax isolated arteries, and microsphere experiments in 5-HT-infused rats suggested that 5-HT increased flow to the splanchnic bed. We hypothesized that 5-HT increased splanchnic flow because of direct venous relaxation; our focus was thus on the superior mesenteric vein (SMV) as an important vein in splanchnic circulation. Real-time RT-PCR, immunohistochemistry and Western analyses supported the predominant expression of the 5-HT_2B_ and 5-HT_7_ receptor in the SMV. The SMV was mounted in tissue baths for measurement of isometric contraction. 5-HT caused a concentration-dependent relaxation of the endothelin-1 (ET-1)-contracted vein. The threshold of 5-HT-induced venous relaxation was significantly lower than for 5-HT-induced venous contraction (∼2 vs. 700 nmol/L, respectively). A series of serotonergic agonists established in their use of receptor characterization was tested, and the following rank order of potency found for agonist-induced relaxation (receptor selectivity): 5-CT (5-HT_1_/5-HT_7_)>5-HT = LP-44 (5-HT_7_)>PNU109291 (5-HT_1D_) = BW723C86 (5-HT_2B_). 8-OH-DPAT (5-HT_1A/7_), CP93129 (5-HT_1B_), mCPBG (5-HT_3/4_), AS19 (5-HT_7_) and TCB-2 (5-HT_2A_) did not relax the isolated vein. Consistent with these findings, two different 5-HT_7_ receptor antagonists SB 269970 and LY215840 but not the 5-HT_2B_ receptor antagonist LY272015 nor the nitric oxide synthase inhibitor LNNA abolished 5-CT-induced relaxation of the isolated SMV. 5-CT (1 *μ*g kg^−1^ min^−1^, sc) also reduced blood pressure over 7 days. These findings suggest that 5-HT directly relaxes the SMV primarily through activation of the 5-HT_7_ receptor.

## Introduction

5-HT was initially described as a vasoconstrictor, given that it elevated the tone of isolated blood vessels in vitro (Page and McCubbin 1953). We discovered that when given chronically over 7–30 days to the conscious rat, 5-HT caused a dose-dependent reduction in blood pressure (Diaz et al. 2008; Tan et al. 2011; Davis et al. 2012, 2013). These long-term hypotensive actions of 5-HT have been a repeatable finding in the hands of a number of different investigators both within and outside of our laboratory (Diaz et al. 2008; Tan et al. 2011; Davis et al. 2012).

We performed microsphere studies in animals treated with 5-HT to determine the vascular beds in which 5-HT functioned to reduce blood pressure. 5-HT, relative to vehicle-infused rats, elevated flow to the splanchnic circulation, including the superior mesenteric vessels (Seitz and Watts 2014). This could occur because 5-HT may directly relax the arterial circulation. However, 5-HT did not cause relaxation when added directly to an isolated artery, either from baseline tone or in a contracted state (Davis et al. 2012). These arteries included the superior mesenteric artery and mesenteric resistance arteries, the vasculature of the tissues in which 5-HT elevated flow in vivo. These experiments excluded mesenteric arteries as directly mediating 5-HT-induced relaxation. Thus, we considered whether the venous circulation could mediate the increase in blood flow through an increase in venous relaxation, ultimately leading to increased capacitance. Venous circulation supports orthostatic hypotension (Bradley and Davis 2003; Frishman et al. 2003), and the vena cava and jugular vein can relax to 5-HT (Trevethick et al. 1984; Sumner et al. 1989; Sumner 1991; Ellis et al. 1995).

In this study, we test the hypothesis that the superior mesenteric vein (SMV) relaxes to 5-HT in a receptor-dependent manner. An important corollary is that 5-HT-induced venous relaxation, if it supports a fall in blood pressure, must be observed in the absence of antagonists of contractile 5-HT receptors because antagonists were not present in animals infused with 5-HT in blood pressure studies. Our focus is on the SMV because of the finding of elevated flow in the splanchnic circulation with chronic 5-HT infusion. Three different molecular techniques – real-time RT-PCR, immunohistochemistry and Western analyses – were used to determine the expression of 5-HT receptor subtypes in the SMV with a focus on those receptors previously associated with vascular relaxation (5-HT_1B_, 5-HT_2B_, 5-HT_7_; Watts et al. 2012). With knowledge gained from these experiments, we moved to the isolated tissue bath to measure isometric contraction. 5-HT caused direct relaxation of the isolated mesenteric vein without having to mask contractile 5-HT receptors. We used a series of serotonergic agonists and antagonists that have been used extensively in receptor characterization, and followed this with a 1 week infusion of the most potent agonist to determine if it could lower blood pressure. This integrative approach is a step in ultimately identifying the 5-HT receptor(s) that mediate 5-HT-induced hypotension.

## Materials and Methods

### Animals

The Michigan State University Institutional Animal Use and Care Committees (IACUC) approved all protocols. Male Sprague–Dawley rats (225–250 g, 8–12 weeks of age, Charles River Laboratories Indianapolis, IN USA) were used.

### Tissue preparation

Naïve rats were anesthetized with pentobarbital (60–80 mg/kg i.p.) and the dissected superior mesenteric artery was placed on a wire (wire through the lumen) on a silastic coated dish filled with physiological salt solution (PSS) containing (mmol/L): NaCl 130; KCl 4.7; KH_2_PO_4_ 1.8; MgSO_4_ · 7H_2_O 1.7; NaHCO_3_ 14.8; dextrose 5.5; CaNa_2_EDTA 0.03, CaCl_2_ 1.6 (pH 7.2). The SMV was embedded in the fat around the artery. Under a microscope, the SMV was carefully dissected out of the fat and placed in the dish. It was then guided onto the wire, cleaned of fat and used in one of the protocols described below. The endothelium was left intact.

### Real-time RT-PCR

From whole SMVs, total RNA was isolated using the MELT Total Nucleic Acid Isolation System and reverse transcribed with Superscript II reverse transcriptase (Invitrogen, Carlsbad, CA). Standard real-time RT-PCR was carried out using a GeneAMP 7500 Real-Time PCR machine (Applied Biosystems, Carlsbad, CA) and FAST SYBR Green PCR Master Mix (Applied Biosystems). Rat primers were purchased from SABiosciences (Frederick, MD): 5-HT_1A_ (RefSeq accession no. NM_012585.1; 191 bp amplicon), 5-HT_1B_ (RefSeq accession no. NM_022225.1; 103 bp amplicon), 5-HT_1D_ (RefSeq accession no. NM_012852.1; 173 bp amplicon), 5-HT_2A_ (RefSeq accession no. NM_017254.1; 191 bp amplicon), 5-HT_2B_ (RefSeq accession no. NM_017250.1; 140 bp amplicon), 5-HT_3A_ (RefSeq accession no. NM_024394.2; 179 bp amplicon), 5-HT_4_ (RefSeq accession no. NM_01285.31; 83 bp amplicon), 5-HT_5A_ (RefSeq accession no. NM_013148.1; 154 bp amplicon), 5-HT_6_ (RefSeq accession no. NM_024365.1; 186 bp amplicon), 5-HT_7_ (RefSeq accession no. NM_022938.2; 99 bp amplicon), and calibrator control (*β*-2 microglobulin; *β*2M) (RefSeq accession no. NM_012512, 128 bp amplicon). PCR conditions were as follows: 95°C for 20 sec followed by 40 cycles of (95°C, 3 sec; 60°C, 30 sec). A standard dissociation curve was run following the above cycle conditions. Each sample was run in duplicate. No template controls (NTC) were run for each primer set.

### Immunohistochemistry

Slides containing sections of paraffin-embedded rat SMV or positive control tissues were dewaxed, antigens retrieved using Unmasking Solution (Vector Laboratories, Burlingame, CA) and taken through a standard protocol. Slides were incubated with 5-HT_1B_ (Abcam, Cambridge, MA; Catalog # ab13896), 5-HT_2B_ (BD Pharmingen, San Diego, CA; Catalog # 556334) or 5-HT_7_ antibody (LS Bio, Seattle, WA; Catalog LS-A7991, 5 *μ*g/mL all antibodies) in the appropriate 1.5% blocking serum. Slides were washed in phosphate-buffered saline and incubated with a species-specific peroxidase-conjugated secondary antibody in 1.5% blocking serum for 30 min, followed by a 30 min incubation in Vectastain Elite ABC Reagent (Vector Laboratories). 3,3-diaminobenzidine/H_2_O_2_ was applied until staining appeared (1–4 min). The slides were counterstained with hematoxylin (Vector Laboratories). Images were captured on a Nikon Eclipse Ti with MMI imaging software (Melville, NY, USA).

### Western blot analysis

Superior mesenteric veins were cleaned, frozen, and then ground into a powder. Homogenation buffer (125 mmol/L Tris [pH 6.8[, 4% SDS, 20% glycerol, 0.5 mmol/L phenylmethylsulfonyl fluoride, 1 mmol/L orthovanadate, 10 *μ*g/mL aprotinin, 10 *μ*g/mL leupeptin) was added and the homogenates were vortexed briefly, sonicated and centrifuged. Supernatants were collected and protein concentration was determined with the BCA protein kit (Sigma (St. Louis, MO, USA), catalog #BCA1). Western analysis of SMV homogenates (50 *μ*g) was performed and proteins transferred to PVDF (5-HT_7_) or nitrocellulose (5-HT_2B_). Positive controls (rat stomach fundus for 5-HT_2B_ receptor [10 *μ*g[, rat brain for 5-HT_7_ receptor [50 *μ*g[) were run in parallel lanes. Blots were then incubated overnight at 4°C with 5-HT_2B_ (1:1000; BD Pharmingen; Catalog # 556334) or 5-HT_7_ primary antibody (1:1000; Abcam; Catalog # ab13898). Following 5-HT receptor antibody incubation, the same blots were reprobed for smooth muscle *α*-actin (1:2000; EMD Chemicals/Calbiochem, Gibbstown, NJ) to ensure equal protein loading. All blots were developed using species-specific HRP-conjugated secondary antibodies and ECL reagents (Amersham/GE Healthcare Life Sciences, Piscataway, NJ).

### Isolated tissue bath

SMV were cleaned and cut into two rings (∼3 mm wide) for measurement of isometric contractile force. Rings were mounted in warmed (37°C) and aerated (95% O_2_, 5% CO_2_) tissue baths (30 mL PSS) on Grass isometric transducers (FT03; Grass instruments, Quincy, MA), connected to an ADInstruments PowerLab (ADInstruments, Colorado Springs, CO). Tissues were placed under optimal resting tension (400 mg) and allowed to equilibrate for 1 h before an initial challenge with a maximal concentration of norepinephrine (10^−5^ mol/L). After this challenge, tissues were washed until tone returned to baseline. Preliminary experiments determined that endothelin-1 (ET-1) caused a stable contraction in the SMV (compared to NE, U46619, PGF2alpha), and thus ET-1 (1 nmol/L) was used as a contractant in experiments, save for when 5-HT was tested from baseline. This concentration of ET-1 is submaximal, achieving ∼40–50% of maximal contraction in SMV. All tissues were not tested for the presence of endothelium because ET-1-induced contraction could not be washed out and reachieved within the normal time course of an experiment. Thus, in separate experiments, the status of the endothelium in veins as typically prepared was tested (described below); these tissues were not used in experiments creating curves to serotonergic agonists. Tissues underwent one of the following protocols:

#### Serotonergic agonists

Once ET-1-induced contraction was stable (∼15 min), either vehicle (volume additions run each time a new agonist was tested) or increasing concentrations of serotonin receptor agonists were added to the bath in a bolus or a cumulative fashion (10^−10^–10^−5^ mol/L). Vehicle additions (volume additions) were not performed for every tissue given that a repeatable response was achieved with *N* = 10. These responses are reported collectively in each panel of Figure 6 and in Table 1. Agonists tested include (primary selectivity in parentheses): 5-HT, 5-carboxamidotryptamine (5-CT; 5-HT_1/7_), 8-OH-DPAT (5-HT_1A/7_), AS19 (5-HT_7_), BW-723C86 (5-HT_2B_), CP 93129 (5-HT_1B_), LP-44 (5-HT_7)_, mCPBG (5-HT_3/4_), PNU109291 (5-HT_1D)_ and TCB-2 (5-HT_2A_). At the end of each experiment, the adenylate cyclase activator forskolin (10^−5^ mol/L) was added if tissues showed no relaxation to a serotonergic agonist. In a few experiments, the SMV was not contracted with ET-1, and 5-HT was added in a cumulative fashion.

**Table 1:** Pharmacological parameters of serotonergic agonists tested in the ET-1 (1 nmol/L)-contracted isolated superior mesenteric vein.

Agonist	Potency (−log EC_50_ [mol/L[)	Efficacy (% ET-1 contraction remaining)
Vehicle (water additions)	–	75.4 ± 4.8
5-CT (5-HT1/7)	8.21 ± 0.20	11.30 ± 4.98^1^
5-HT	7.48 ± 0.55	27.00 ± 13.8^1^
LP-44 (5-HT7)	7.55 ± 0.18	16.30 ± 5.80^1^
PNU109291 (5-HT1D)	6.26 ± 0.16	22.00 ± 9.50^1^
BW723C86 (5-HT2B)	6.06 ± 0.46	47.20 ± 10.40^1^
mCPBG (5-HT3/4) (*N* = 6)	4.71 ± 0.14	55.60 ± 5.40^1^
8-OH-DPAT (5-HT1A/7) (*N* = 6)	NC	79.08 ± 12.45
CP93129 (5-HT1B) (*N* = 4)	NC	95.20 ± 4.20
AS19 (5-HT7) (*N* = 6)	NC	77.40 ± 9.90
TCB-2 (5-HT2A) (*N* = 4)	NC	135.9 ± 17.70

Data are presented as means ± SEM for the number of animals presented in graphical figure. NC, not converged, could not estimate an EC_50_ value.

1 Significant difference from effect observed in vehicle-incubated tissues.

#### Serotonergic antagonists

Tissues were incubated with either vehicle (water, 0.01% Dimethylsulfoxide (DMSO)), LY272015 (5-HT_2B_), LY215840 (5-HT_7_), or SB269970 (5-HT_7_) for 45 min prior to establishing contraction with ET-1 (1 nmol/L). 5-CT (10^−10^–10^−5^ mol/L) was added in a cumulative fashion. If tissues did not relax, forskolin (10^−5^ mol/L) was added to test their ability to relax.

#### ACh versus 5-CT comparison and NOS

In a few experiments, the status of the endothelium, as reflected by the relaxation to ACh (1 *μ*mol/L), was examined. Relaxation to 5-CT (1 *μ*mol/L) was compared directly. Tissues were contracted with ET-1 (1 nmol/L) and exposed to either agonist. Tissues were washed for ∼5 h (wash every 15 min), and then recontracted to ET-1 and challenged with the agonist not examined previously. The order of agonists was randomized in experiments. In other experiments, tissues were incubated with vehicle or the nitric oxide synthase (NOS) inhibitor LNNA (100 *μ*mol/L) for 45 min prior to establishing contraction with ET-1 (1 nmol/L). 5-CT (10^−10^–10^−5^ mol/L) was then added in a cumulative fashion.

### Telemetry and pump implantation

Radiotelemeter transmitters (Data Sciences International, MN(St. Paul, MN, USA)) with attached catheters with pressure-sensing tips were implanted subcutaneously through a 1–1.5-cm incision in the left inguinal area while rats were under isoflurane anesthesia. Catheters were introduced into the left femoral artery 3–5 mm distal to the level of the peritoneal wall, and the tip was advanced to the abdominal aorta. Rats were allowed 3–4 days to recover postoperatively, and then 3–4 days of baseline measurements were made. Mean arterial pressure, pulse pressure, and heart rate were recorded throughout the duration of the study. Seven to ten days after radiotelemeter placement, osmotic pumps with a release rate of 10.0 *μ*L/h and duration of 7 days (Model 2ML1, Alzet Osmotic Pumps (Cupertino, CA USA)) were implanted subcutaneously between the scapulae. Two groups were used: a group receiving vehicle and a group receiving 5-CT (1 *μ*g/kg/min, s.c.). Vehicle was 1% ascorbate (antioxidant) in sterile saline, pH balanced to between 6 and 7.

### Materials

Acetylcholine chloride, 5-CT maleate, 5-HT creatinine sulfate, forskolin, and norepinephrine hydrochloride were obtained from Sigma Chemical Company (St. Louis, MO). 8-OH-DPAT, AS-19, BW723C86, CP93129, LP-44, LY215840, LY272015, mCPBG, PNU109291, SB269970, and TCB-2 were purchased from Tocris (R& D systems, Minneapolis, MN). ET-1 (1-21) was purchased from Bachem (Torrance, CA).

### Statistical analysis

All quantitative data are reported as means ± SEM for number of animals in parentheses. 5-HT receptor mRNA expression is expressed relative to *β*2M. For immunohistochemistry experiments, sections from a minimum of four animals were used. Adjustments in brightness and contrast were made to the whole panel of an image (photograph or Western image), not a portion. Values for Western analyses were densitized in ImageJ (http://imagej.nih.gov/ij/), and are reported as arbitrary densitometry unit relative to *α*-actin densitometry units. For isometric contractile studies, relaxation is reported as a percentage of initial contraction to a half-maximal concentration of ET-1 as this is the contraction against which relaxation occurs. Contraction is reported as a percentage of initial contraction to NE. Agonist potencies were calculated using a nonlinear regression (curve fit) within GraphPad Prism 6.0 (La Jolla, CA), and are reported as −log EC_50_ values [mol/L[. Maximums (contraction or relaxation) are reported as the maximal effect achieved. Where a maximal response was not achieved, the actual potency (EC_50_ value) was considered equal or greater than the reported value. Blood pressures and heart rate are reported as differences from baseline measures in mmHg and beats per minute, respectively. Repeated measures two-way analysis of variance followed by the Bonferroni post hoc test was used to compare concentration–response curves. In all cases, *P *<* *0.05 was considered significant.

## Results

### Multiple 5-HT receptors are presented in the SMV

**Figure 1:**
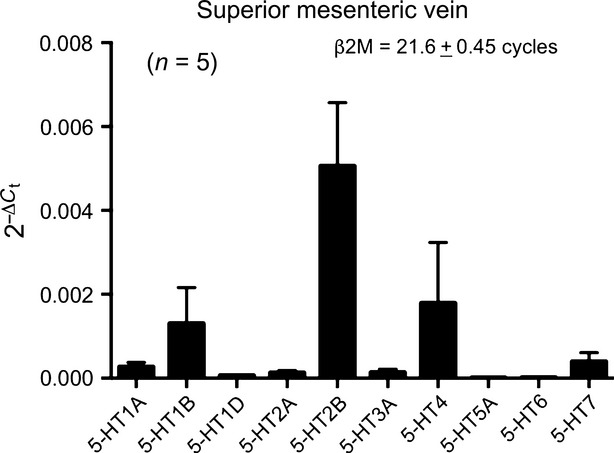
Real-time RT-PCR for 5-HT receptor mRNA in the superior mesenteric vein. The data are reported relative to *β*2-microglobulin (*β*2M; *C*_*T*_ = 21 ± 0.45 cycles). Bars represent means ± SEM for number of animals in parentheses.

Real-time RT-PCR of the whole mesenteric vein demonstrated the presence of mRNA for several 5-HT receptors, and message for three of these receptor subtypes was notable. The 5-HT_1B_, 5-HT_2B_, and 5-HT_7_ receptor mRNA was observed consistently (Fig. 1). Lesser 5-HT_1A_ mRNA was observed, and the signal for 5-HT_4_ receptors was highly variable. Expression of mRNA for those receptors associated with vascular contraction was also present, though at low levels (5-HT_1D_, 5-HT_2A_ receptor).

**Figure 2:**
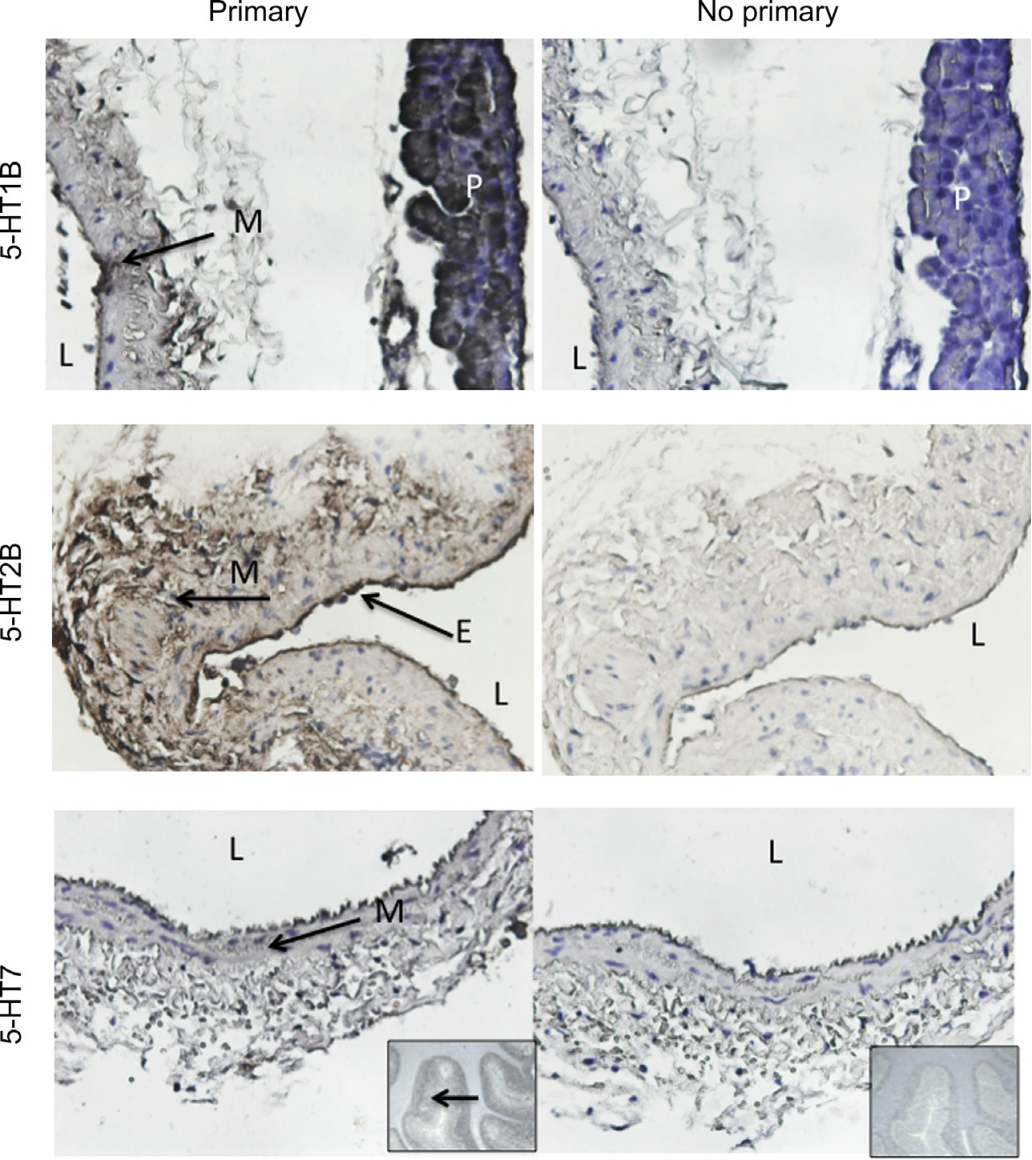
Images from immunohistochemical experiments aimed to detect 5-HT_1B_ (top), 5-HT_2B_ (middle) and 5-HT_7_ (bottom) receptor in the isolated superior mesenteric vein. Images are representative of a minimum of four (4) different animals; arrows point to regions of interest. The left column depicts images of sections incubated with the specific 5-HT primary antibody, and the right column sequential sections incubated without the primary antibody. P, pancreatic tissue; E, endothelium; M, media. Brain sections were used as a positive control for 5-HT_7_ receptor, and these images are inset into the bottom row with experimental images.

**Figure 3:**
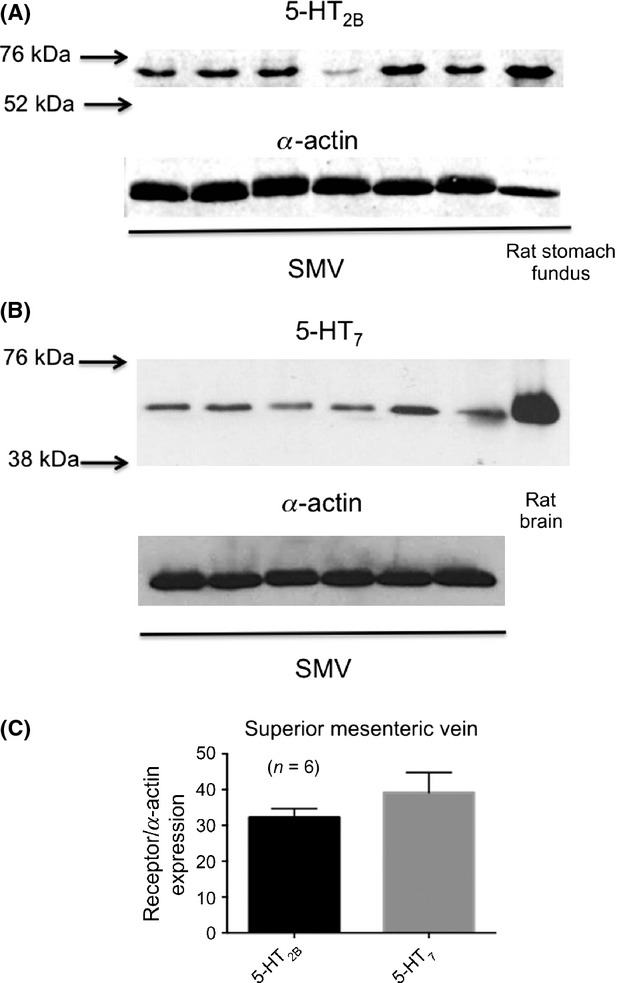
Western analyses of 5-HT_2B_ (A) and 5-HT_7_ (B) receptor in the isolated rat superior mesenteric vein. Each lane represents a different animal, and lanes on the far right hand side are lanes loaded with appropriate positive control (rat stomach fundus for 5-HT_2B_, rat brain for 5-HT_7_). Alpha actin was used as a loading control for the superior mesenteric vein. C depicts densitometric analyses of 5-HT_2B_ and 5-HT_7_ receptor expression relative to alpha actin expression. Bars represent means ± SEM for number of animals in parentheses.

In the SMV, immunohistochemical analyses for the protein of the 5-HT receptors associated with vascular relaxation are shown in Figure 2 (arrows point to areas of interest). The strongest signal (difference in intensity between the sections incubated with and without primary antibody) was observed with an antibody directed against the 5-HT_2B_ receptor (middle panel). This significant expression was observed in an area consistent with the endothelial cell (E) as well as through the muscular medial wall (M). Weakly positive staining was also observed for the 5-HT_7_ receptor in the venous media (bottom); brain sections stained strongly with this same concentration of antibody (inset). Staining for the 5-HT_1B_ receptor was weak (top), but the ability of the antibody to recognize the 5-HT_1B_ receptor was validated by the positive staining of the pancreatic tissue (P) that accompanied the venous section. These general findings were corroborated with Western analyses of whole tissue homogenates of cleaned SMV. The 5-HT_2B_ receptor signal was observed as a 72 kDa band that migrated with that of the positive control, the stomach fundus (Fig. 3A); this was the primary band on this blot. The 5-HT_7_ receptor was also expressed in the SMV, with a MW consistent with that observed in the rat brain (∼52 kDa). The band on the 5-HT_7_ blot was the only band detected. Image J allowed for visualization of a clean, symmetrical peak with a discrete area under the curve for all samples in the 5-HT_2B_ and 5-HT_7_ receptor blots, and the alpha actin signal was readily quantified for normalization. Densitometry of these supports a quantitatively similar expression of the 5-HT_2B_ and 5-HT_7_ receptor in the SMV. Western analyses of the 5-HT_1B_ receptor were not pursued because of the weak immunohistochemical signal in the SMV. These three approaches – real-time RT-PCR, immunohistochemistry and Western analyses – support the 5-HT_2B_ and 5-HT_7_ receptor as receptors of interest.

### 5-HT contracts and relaxes the isolated SMV

**Figure 4:**
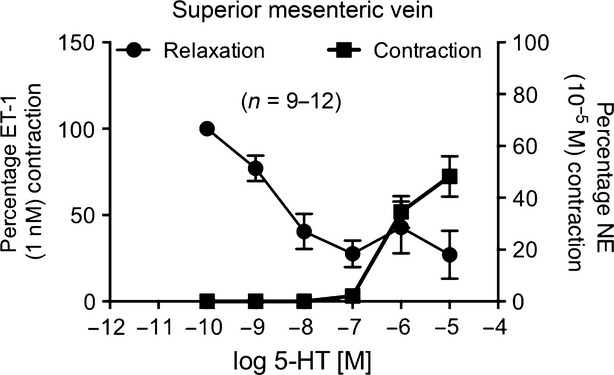
Effect of 5-HT in isolated superior mesenteric vein when 5-HT was added at baseline (squares; contraction) or after ET-1-(1 nmol/L) induced contraction was established (circles; relaxation). Contraction is reported as a percentage of initial contraction to NE (151 ± 22 mg) and relaxation is reported as the percentage of ET-1-induced contraction (146 ± 9.3 mg) remaining in the presence of 5-HT. Points represent means ± SEM for number of animals in parentheses.

**Figure 5:**
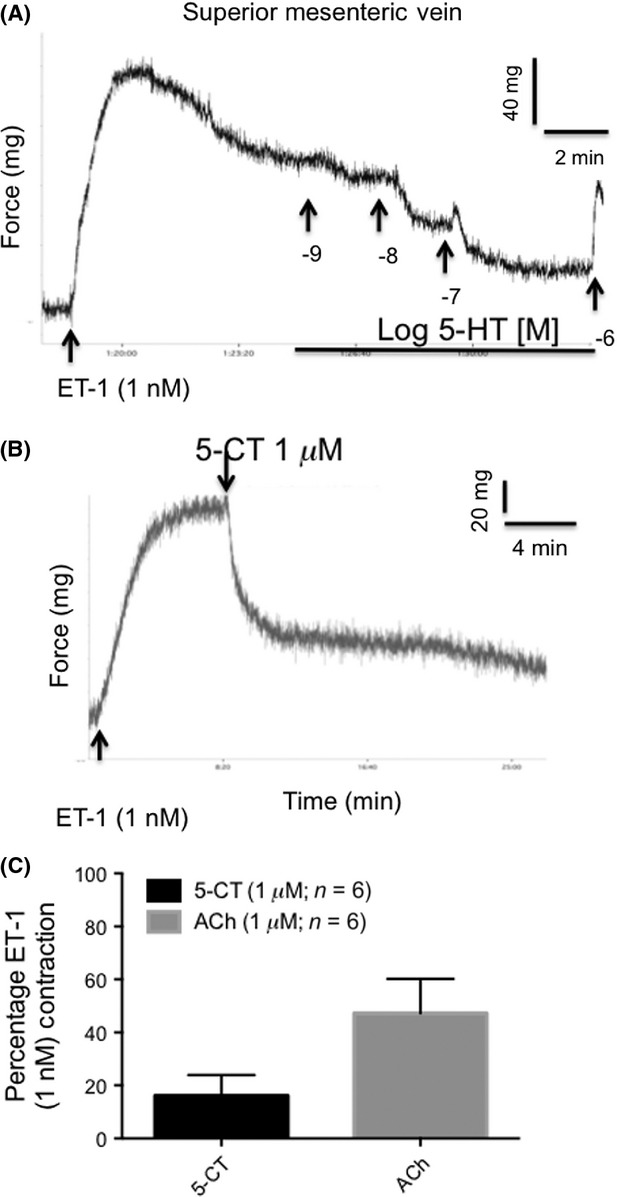
Representative tracing of isolated rat superior mesenteric vein to 5-HT (A) or bolus of 5-CT (B). Response is reported in milligrams, and scale bars for force and time are in the corner of each tracing. ET was added at the first arrow, and then either 5-HT (A) or 5-CT (B) introduced. (C) depicts the comparative magnitude of relaxation of the endothelium-dependent agonist ACh to 5-CT-induced relaxation in ET-1 contraction superior mesenteric veins. Bars represent means ± SEM for the number of animals in parentheses.

Figure 4 depicts the dual actions of 5-HT in the SMV. From baseline (no agonist-induced tone), 5-HT caused a concentration-dependent contraction. The potency of this contraction was ∼700 nmol/L (−log EC_50_ value [mol/L[ = 6.22 ± 0.01). When the mesenteric vein was contracted before addition of 5-HT, the outcome changed. 5-HT caused a concentration-dependent relaxation with significantly higher potency (−log EC_50_ [mol/L[ = 8.60 ± 0.25 or 2.5 nmol/L) when compared with its contractile potency. A representative tracing of this relaxation is shown in Figure 5A. Experiments were carried out in the absence of antagonists of 5-HT receptors that mediate contraction, and thus in concentrations higher than 100 nmol/L 5-HT, contraction was observed. We show the effect of a high concentration of 5-CT (1 *μ*mol/L) on ET-1-induced contracted vein (Fig. 5B) given that this agonist would prove to be potent and without direct contractile effect. In experiments in which 5-CT was given as a bolus like this, 5-CT (1 *μ*mol/L) relaxed ET-1-induced contraction to 16 ± 7% of original contraction (Fig. 5C). By comparison, ACh (1 *μ*mol/L) relaxed the same tissues contracted with the same concentration of ET-1 to 47 ± 13% of the original contraction (Fig. 5C). The magnitude of ET-1-induced contraction was not different in these two different challenges (5-CT = 161 ± 60 mg; ACh = 155 ± 35 mg, *P* < 0.05). In separate experiments, the NOS inhibitor LNNA (100 *μ*mol/L) did not reduce the maximal relaxation stimulated by 5-CT (1 *μ*mol/L; Vehicle = 20 ± 7.9 ET-1 contraction; LNNA = 29.8 ± 10.6% ET-1 contraction, *P* > 0.05). These experiments provided the impetus to perform the following pharmacological experiments.

### Pharmacological identification of 5-HT relaxant receptor in SMV

**Figure 6:**
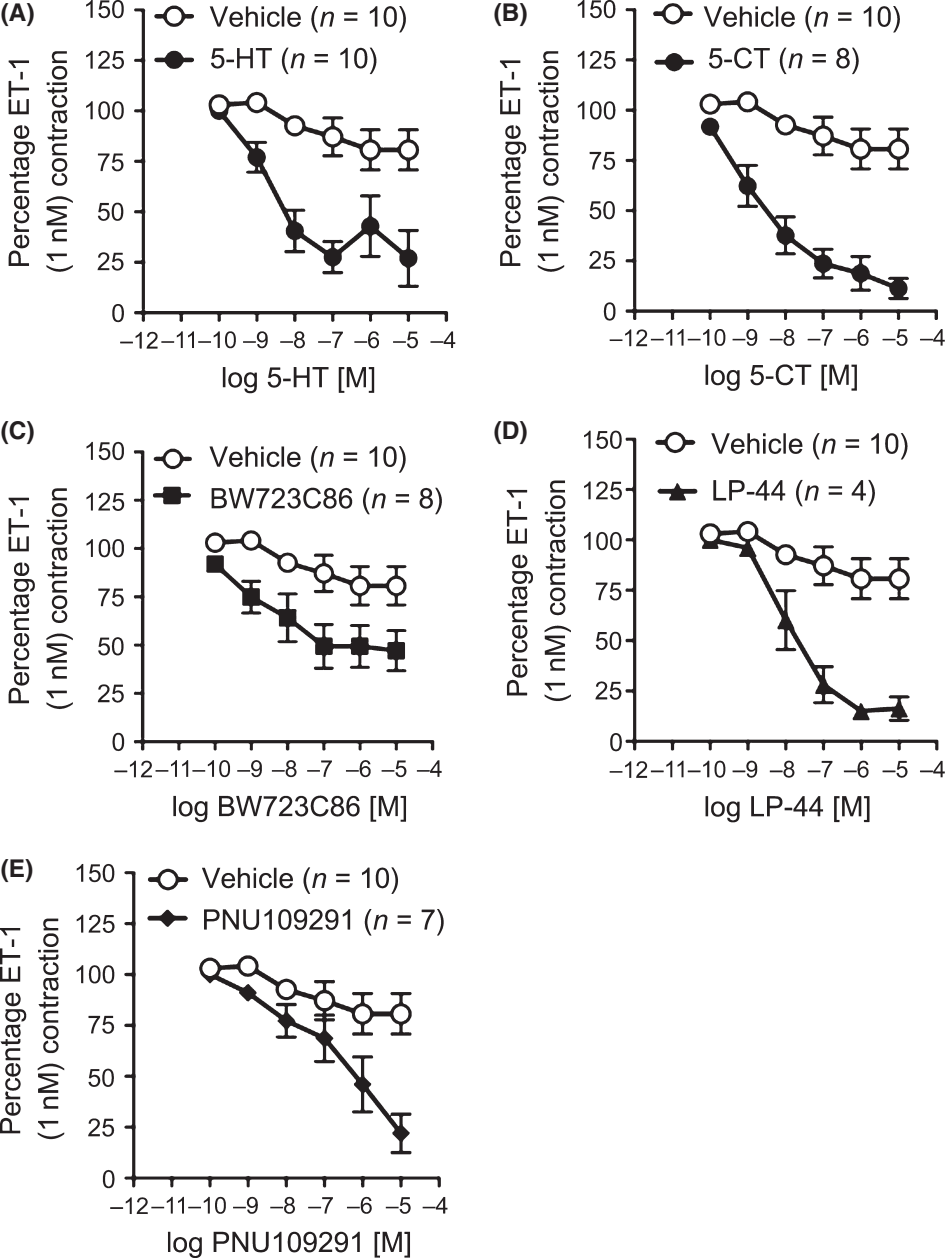
Effects of efficacious serotonergic receptor agonists on ET-1 (1 nmol/L)-induced contraction in the isolated superior mesenteric vein. The Vehicle curve is repeated in each graph for comparison. Points represent means ± SEM for number of animals indicated in parentheses. (A) 5-HT, (B) 5-CT, (C) BW723C86, (D) LP-44, (E) PNU109291.

A series of serotonergic agonists was tested for their ability to relax the ET-1-contracted SMV. Agonists which caused a concentration-dependent, efficacious relaxation are shown individually in Figure 6. The vehicle curve, where the same volume of agonist additions was added, was constructed to be able to account for loss of ET-1-induced contraction over time and volume addition, but was not run for every vein. Over the approximately 90 min these curves took to construct, ∼24% of ET-1-induced contraction was lost; this collective curve was placed on each individual graph. Table 1 shows pharmacological parameters of serotonergic agonists in stimulating venous relaxation. 5-CT (Fig. 6B) was more potent than 5-HT (Fig. 6A), and did not cause the contraction at higher concentrations as did 5-HT. The 5-HT_2B_ partial agonist BW723C86 caused modest relaxation (Fig. 6C), whereas the putative 5-HT_7_ receptor agonist LP-44 (Fig. 6D) was similarly efficacious to 5-CT. PNU109291 (5-HT_1D_ agonist) was least potent in causing relaxation (Fig. 6E).

In contrast to these agonists, those with affinity for the 5-HT_1B_ receptor (CP93129), 5-HT_2A_ receptor (TCB-2), 5-HT_3/4_ receptor (mCPBG), and 5-HT_1A/7_ receptor (8-OH-DPAT) did not cause a relaxation that was of a magnitude greater than that caused by vehicle; pharmacological parameters for these agonists are reported in Table 1. These agonists were chosen given that the mRNA for their receptors was expressed in detectable amounts (Fig. 1), and thus we needed to determine whether the receptors could mediate relaxation. AS19, another putative 5-HT_7_ receptor agonist, did not relax the SMV. The tissues in which these serotonergic agonists were tested relaxed completely to forskolin (10 *μ*mol/L), validating their intrinsic ability to relax. Collectively, these results are most consistent with 5-HT_2B_ and/or 5-HT_7_ receptors mediating relaxation.

**Figure 7:**
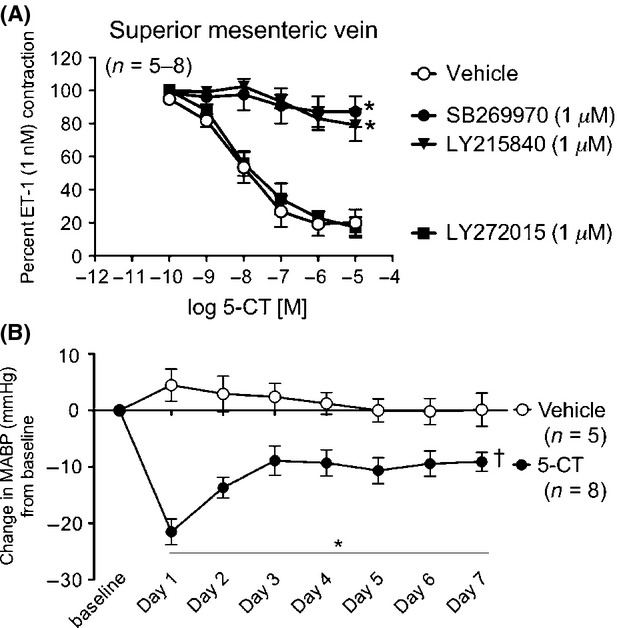
(A). Effect of 5-HT_2B_ (LY272015) and 5-HT_7_ (SB269970 and LY215840) receptor antagonists on 5-CT relaxation stimulated in the ET-1 (1 nmol/L)-contracted isolated superior mesenteric vein. (B). Ability of 5-CT to lower blood pressure of the conscious rat. Data are graphed as a change from baseline mean arterial blood pressure (MABP) where vehicle MABP = 98.4 ± 2.2 mm Hg, 5-CT MABP = 99.4 ± 2.4 mmHg. Points represent means ± SEM for number of animals indicated in parentheses. *Signify significant differences from vehicle, †from baseline values.

To corroborate these findings, we examined the ability of three different 5-HT receptor antagonists to block relaxation. In these experiments, 5-CT was used as the model agonist given its inability to cause the biphasic response stimulated by 5-HT in ET-1-contracted veins. LY272015 (1 *μ*mol/L), a 5-HT_2B_ receptor antagonist, did not shift or reduce 5-CT-induced relaxation (Fig. 7A). A 100-fold lower concentration of LY272015 rightward shifted 5-HT-induced contraction ∼100-fold in the isolated rat stomach fundus, evidence supporting our use of this compound as an antagonist of 5-HT_2B_ receptors (Russell et al. 2002). In contrast, two different 5-HT_7_ receptor antagonists, LY215840 and SB269970, abolished 5-CT-induced relaxation (Fig. 7A). These findings lend strong support to the 5-HT_7_ receptor mediating agonist-induced serotonergic relaxation in the SMV.

### 5-CT lowers blood pressure chronically

With the knowledge that 5-CT was a potent relaxant of the SMV, we next infused this agonist in conscious rats. Dosing studies supported use of 1 *μ*g kg^−1^ min^−1^ 5-CT in the Alzet pump as causing the greatest fall in blood pressure; using higher concentrations were not necessary. Figure 7B depicts the fall in blood pressure during a 1-week infusion of 5-CT. Mean arterial blood pressure fell over 20 mmHg during the first day of infusion, never recovering to levels equivalent to control or those infused with vehicle. Heart rate elevated reflexively during the time of blood pressure fall, but recovered by day 4 (not shown). These studies are consistent with our in vitro findings.

## Discussion

In the anesthetized rat, acutely injected 5-HT causes a triphasic response consisting of a fast depressor, slower pressor and then a long depressor response (Dalton et al. 1986). Several laboratories have suggested that the long (e.g., minutes after 5-HT injection) depressor response is due to activation of 5-HT_7_ receptors in the arterial circulation (Terrón 1997; De Vries et al. 1999; Hedlund and Sutcliffe 2004). Our laboratory has infused 5-HT chronically (7–30 days) and observed a hypotension that is long lasting; these were the first studies to demonstrate a chronic ability of 5-HT to reduce blood pressure (Diaz et al. 2008; Tan et al. 2011; Davis et al. 2012). We have yet to identify the mechanism by which this occurs. Presently, we provide the first evidence that mesenteric veins, which handle a substantial portion of cardiac output (Granger and Kvietys 1981), relax to low concentrations of this autocoid. The importance of the veins in blood pressure regulation was reviewed by Fink (2009).

### Identification of functional 5-HT receptors in the SMV

We performed real-time RT-PCR first to verify the presence of 5-HT receptors and the subtypes expressed. Significant signal for the 5-HT_1B_, 5-HT_2B_, and 5-HT_7_ receptor was observed. The 5-HT_4_ signal was highly variable, and the potency of mCPBG in relaxing the isolated vein was low and not consistent with interaction with the 5-HT_3_ or 5-HT_4_ receptor (5-HT affinity at 5-HT_4_ receptor is 3–10 nmol/L; http://www.iuphar-db.org/DATABASE/ObjectDisplayForward?familyId=1&objectId=9&familyType=RECEPTOR). The 5-HT_3/4_ receptors have not been observed as participants in vascular function except for one report (Cocks and Arnold 1992). Similarly, 5-HT_1B_ receptor expression was not strong, and use of CP93129 as a 5-HT_1B_ receptor agonist in isolated tissue bath experiments supported either the lack of protein expression and/or lack of function of the 5-HT_1B_ receptor if expressed. We did not pursue these two receptor subtypes further. The molecular analyses performed allowed us to focus on the 5-HT_2B_ and 5-HT_7_ receptor.

These two receptor subtypes have the strongest evidence that support their involvement in vascular relaxation. The 5-HT_2B_ receptor was originally identified in the rat stomach fundus (Kursar et al. 1994) as a contractile receptor, but has since been localized to the endothelium where it mediates vascular relaxation (Ellis et al. 1995). The 5-HT_2B_ receptor is upregulated in arterial smooth muscle and mediates 5-HT-induced contraction in arteries from deoxycorticosterone acetate (DOCA) salt hypertensive rats, a finding that counters the idea that 5-HT interacts with this receptor to cause a hypotension (Watts et al. 1995). The 5-HT_2B_ partial agonist BW723C86 caused concentration-dependent relaxation and was partially efficacious compared to 5-HT or 5-CT. The potency of BW723C86 was not consistent with interaction with purely 5-HT_2B_ receptors, and the finding that 5-CT-induced relaxation could not be antagonized by the 5-HT_2B_ receptor antagonist LY272015 suggests the 5-HT_2B_ receptor is minimally involved. The concentration of LY272015 used should saturate the 5-HT_2B_ receptor, but neither the potency nor efficacy of 5-CT was changed by LY272015. Given these findings, the 5-HT_2B_ receptor appears to play a minimal role in the SMV, but we cannot completely exclude its potential role because BW723C86 did cause a relaxation greater than vehicle, and the 5-HT_2B_ receptor is clearly present in the mesenteric vein.

### Evidence for the 5-HT_7_ receptor mediating relaxation in the SMV

Several pieces of evidence support a focus on the 5-HT_7_ receptor in the SMV. Real-time RT-PCR, immunohistochemistry and especially Western analyses validate the presence of this 5-HT receptor in the SMV. Strong pharmacological evidence supports the presence of a functional 5-HT_7_ receptor in the vein. 5-CT, which has significant affinity for the 5-HT_7_ receptor, 5-HT and LP-44 all relaxed the ET-contracted vein. Furthermore, two different 5-HT_7_ receptor antagonists abolished 5-CT-induced relaxation. Literature supports that both LY215840 and SB269970 can act competitively and noncompetitively in isolated tissues and our finding of significant reduction of the response at a 1 *μ*mol/L concentration of 5-HT_7_ receptor antagonists is consistent with published work (Cushing et al. 1996; Hagan et al. 2000; Tuladhar et al. 2003). One experimental outcome is in disagreement with these findings. AS19, another 5-HT_7_ receptor agonist, lacked potency and efficacy in relaxing the ET-contracted vein. Our knowledge of the pharmacology of AS19 is not robust, and at least one study suggests that AS19 lacks expected agonist activity at the 5-HT_7_ receptor (Wang et al. 2010). In our searches, virtually all of the published studies using AS19 have been in vivo studies, not in isolated blood vessels. Thus, we can only speculate that there must be a difference in the intrinsic efficacy of LP44 and AS19 in the SMV.

Another concern is why 8-OH-DPAT, the 5-HT_1A_ receptor agonist with recognized affinity for the 5-HT_7_ receptor, was not a potent and/or efficacious agonist in the mesenteric vein. We propose two explanations. The first is that 8-OH-DPAT has lower affinity for the 5-HT_7_ receptor than 5-CT and 5-HT. 5-CT, LP44, and AS19 all have subnanomolar affinity, followed by 5-HT with nmol/L affinity, for the 5-HT_7_ receptor (http://pdsp.med.unc.edu/pdsp.php). 8-OH-DPAT has anywhere from 35 to 1995 nmol/L affinity (Ki) at the rat 5-HT_7_ receptor, making 8-OH-DPAT a less potent agonist than 5-HT and 5-CT. The rank order of agonists in causing venous relaxation in our study was consistent with the profile of the 5-HT_7_-receptor-mediated relaxation of the porcine oviduct (Inoue et al. 2003), giving us confidence in stating that the 5-HT_7_ receptor is likely involved in the venous relaxation. The second explanation is that 8-OH-DPAT has been described as a partial agonist of 5-HT_7_ receptors, not a full agonist. Several independent groups provide data supporting that 8-OH-DPAT is a partial agonist versus 5-HT and 5-CT in second messenger production, primarily adenylate cyclase activation (Thomas et al. 1999; Wood et al. 2000; Krobert et al. 2001; Rauly-Lestienne et al. 2007). The combination of a lower affinity for and efficacy at the 5-HT_7_ receptor could result in 8-OH-DPAT having a poor ability in relaxation the SMV.

Importantly, we demonstrated that a long-term (1 week) infusion of 5-CT, which can act as a 5-HT_7_ receptor agonist, causes a fall in blood pressure directly. These findings are consistent with the hypothesis that the 5-HT_7_ receptor may mediate the hypotension, but this remains to be proven. The function of the 5-HT_7_ receptor to serve a hypotensive function was suggested by De Vries et al. (1999) and Terrón (1997), but our studies are novel and different in two ways. First, the rats studied presently were conscious and freely moving, and not vagotomized or pithed, not anesthetized and not treated with receptor antagonists to mask contractile receptors. Second, administration of 5-HT was not acute (minutes) but over the course of 7 days. This, then, is an all together different model than has been used previously. Our present findings are powerfully supportive of the ability of 5-HT to reduce blood pressure in the long term.

5-HT and/or 5-CT relax the rat meninges (Martinez-Garcia et al. 2011), pig pulmonary artery (Jahnichen et al. 2005), guinea pig mesenteric lymphatics (Chan and von der Weid 2003), canine external carotid (Centurion et al. 2000; Villalon et al., 2001), pig pial vein (Ishine et al. 2000), and canine cerebral artery (Terron and Falcon-Neri 1999). In some but not all of these studies, contractile 5-HT receptors (5-HT_2A_, 5-HT_1D_) had to be blocked before relaxation was visualized. This was more so the case in the arterial versus venous studies, which lends support to the idea that 5-HT given in vivo could, in a naïve state, interact with the venous circulation to cause blood pressure to fall. Importantly, venous relaxation to 5-HT has been reported in a number of different species, including goat (Chand 1981), sheep (Cocks and Arnold 1992; Zhang et al. 1995), rat (Ellis et al. 1995), guinea pig (Gupta 1992), pig (Ishine et al. 2000; Komore et al. 1989; Sumner 1991, Sumner et al. 1989; Trevethick et al. 1984), cynomolgus monkey (Leung et al. 1996), and rabbit (Tsuru et al. 1998). None of these reports study the mesenteric veins. However, they do support the general concept that the venous vasculature relaxes to 5-HT; we have yet to find reports of 5-HT-induced relaxation in veins from human and this is of significant interest.

### Limitations and looking forward

We acknowledge a few limitations of this work. First, we used 5-CT as a model agonist. 5-CT has high affinity (sub to low nmol/L) for the 5-HT_1A_, 5-HT_1B_, 5-HT_1D_, 5-HT_5_, and the 5-HT_7_ receptors, lesser affinity for the 5-HT_2B_, 5-HT_2C_, and 5-HT_6_ receptors and low affinity for the 5-HT_2A_, 5-HT_3_, and 5-HT_4_ receptors (http://pdsp.med.unc.edu/, 5-CT as test ligand). 5-HT_5_ and 5-HT_6_ receptors are not known for expression in the vasculature, and our isometric work suggests that the 5-HT_1A_, 5-HT_1B_, and 5-HT_1D_ receptor in the vein do not mediate relaxation. Both agonists – 5-HT and 5-CT – demonstrated concentration-dependent relaxation, but 5-CT was used as a model agonist because it did not cause contraction in high concentrations in the ET-1-contracted vein. Importantly, we present data that 5-CT was able to reduce blood pressure, consistent with venous relaxation. We recognize that this consistency does not prove that 5-CT caused a reduction in blood pressure through an increase in venous capacitance. Second, we focused on the SMV as the primary vessel of interest. This was specifically chosen because our previously published microsphere studies pointed to the splanchnic bed as the site of increased flow (Seitz and Watts 2014), and the arteries of this bed did not relax directly to 5-HT. Third and finally, we have not directly investigated whether the endothelial cell is necessary to agonist-induced relaxation, and it is currently unclear as to whether this cell type is important to 5-HT-induced relaxation. We studied the SMV in which the endothelial layer was not modified (no rubbing, minimal touching) because our goal was to use a model that reflected the in vivo situation in which the endothelium is intact. We did attempt to remove the endothelial layer mechanically from the SMV, but damaged the smooth muscle layers upon doing so. In a different approach, we showed that 5-CT caused relaxation in tissues in which ACh caused relaxation, but the NOS inhibitor did not reduce maximal relaxation to 5-CT. These data suggest that NOS, well known for activity in the endothelial cell, is minimally important to 5-CT-induced relaxation, but does not allow us to conclude whether the endothelial cell is unnecessary for 5-CT-induced relaxation.

Several studies using compounds that inhibit the serotonin transporter (SERT) inhibitors are associated with hypotension (Alexandrino-Silva et al. 2009; reviewed in Watts et al. 2012), suggesting that a buildup of circulating 5-HT may promote the hypotension. At the same time, fluoxetine, the first SERT inhibitor, has been used to treat orthostatic hypotension (Grubb et al. 1994) and can cause hypertension in rodent models (Tsai and Lin 1986; Lazartigues et al. 2000). It would be particularly interesting to examine the role of 5-HT in orthostatic hypotension in patients with autonomic failure, given that their physiological feedback systems are negated, and thus one might obtain a purer read out of what the circulation, independent of the sympathetic nervous system, can do. Finally, the idea of upregulation of the venous 5-HT_7_ receptor or sensitivity in disease should be considered. In the DOCA-salt model, circulating free 5-HT is elevated, and the DOCA-salt hypertensive rat displays a profound (>50 mmHg) hypotension to 5-HT when it was given chronically (Diaz et al. 2008). This particular hypertension model has at least one adapative mechanism that attempts to lower blood pressure. Calcitonin gene related peptide and its receptors play a compensatory role such that when the receptor is antagonized in the DOCA-salt rat, blood pressure rises even higher (Supowit et al. 1997). We speculate upregulation of the 5-HT_7_ receptor in veins could function in a similar manner.

In conclusion, we present the original finding that the SMV possesses a highly sensitive 5-HT_7_ receptor that mediates venous relaxation without requiring blockade of contractile receptors. We argue this is likely the primary receptor mediating relaxation to 5-HT, but cannot exclude the potential involvement of the 5-HT_2B_ receptor. This work elevates the important of the venous circulation in control of blood pressure, and provides a better direction for understanding the mechanisms by which 5-HT lowers blood pressure.

## Abbreviations

5-CT: 5-carboxamidotryptamine
5-HT: 5-hydroxytryptamine, serotonin
8-OH-DPAT: (±)-8-Hydroxy-2-dipropylaminotetralin
AS19: (2*S*)-(+)-5-(1,3,5-Trimethylpyrazol-4-yl)-2-(dimethylamino)tetralin
BW723C86: *α*-methyl-5-(2-thienylmethoxy)-1*H*-indole-3-ethanamine
CP93129: 1,4-Dihydro-3-(1,2,3,6-tetrahydro-4-pyridinyl)-5*H*-pyrrol[3,2-*b*[pyridin-5-one
LNNA: NG-nitro-l-arginine
LY215840: [8*β*(1*S*,2*R*)[-*N*-(2-Hydroxycyclopentyl)-6-methyl-1-(1-methylethyl)-ergoline-8-carboxamide
LY272015: 1-[(3,4-Dimethoxyphenyl)methy[-2,3,4,9-tetrahydro-6-methyl-1*H*-pyrido[3,4-*b*[indole
mCPBG: 1-(3-Chlorophenyl)biguanide
SB269970: (2*R*)-1-[(3-Hydroxyphenyl)sulfonyl[-2-[2-(4-methyl-1-piperidinyl)ethyl[pyrrolidine
TCB-2: (4-Bromo-3,6-dimethoxybenzocyclobuten-1-yl)methylamine
